# Supplementary material for: Comparative genomics provides new insights into the diversity, physiology, and sexuality of the only industrially exploited tremellomycete: Phaffia rhodozyma
Source: BMC Genomics. 2016 Nov 9;17:901. doi: 10.1186/s12864-016-3244-7 (PMC5103461; doi:10.1186/s12864-016-3244-7)
Supplement: Additional file 6: — List of orphan genes with links to PFAM (related to Additional file 1: Table S1). (ZIP 1428 kb) [file 12864_2016_3244_MOESM6_ESM.zip › PFAM_UNIQUE/G02822_P.html]

#                                                                            --- full sequence --- -------------- this domain -------------   hmm coord   ali coord   env coord
  
# target name        accession   tlen query name           accession   qlen   E-value  score  bias   #  of  c-Evalue  i-Evalue  score  bias  from    to  from    to  from    to  acc description of target
  
#------------------- ---------- ----- -------------------- ---------- ----- --------- ------ ----- --- --- --------- --------- ------ ----- ----- ----- ----- ----- ----- ----- ---- ---------------------
  
G02822\_P             -           1056 Phostensin           PF13914.1    132       4.8    6.0   6.5   1   2      0.46   1.4e+03   -2.0   0.4    20    44   504   528   451   542 0.68 - Phostensin PP1-binding and SH3-binding region  
G02822\_P             -           1056 Phostensin           PF13914.1    132       4.8    6.0   6.5   2   2   0.00036       1.1    8.1   1.1    43   110   668   737   635   750 0.69 - Phostensin PP1-binding and SH3-binding region
